# Meta-analysis of stage-specific *Calanus finmarchicus* vertical distribution in relation to hydrography and chlorophyll in the North Atlantic

**DOI:** 10.1093/plankt/fbaf019

**Published:** 2025-06-21

**Authors:** Eva Chamorro, Kanchana Bandara, Kaja Balazy, Cecilie Broms, Malin Daase, Eilif Gaard, N Sören Häfker, Xabier Irigoien, Slawomir Kwasniewski, Martin Lindegren, Anders Mosbech, Bettina Meyer, Hildur Petursdottir, Emilia Trudnowska, Sünnje L Basedow

**Affiliations:** Department of Arctic and Marine Biology, Faculty of Biosciences, Fisheries and Economics, UiT The Arctic University of Norway, Framstredet 39, Tromsø 9019, Norway; Akvaplan-niva AS, Fram Centre—High North Research Centre for Climate and the Environment, Postboks 6606, Stakkevollan, Tromsø 9296, Norway; Institute of Oceanology Polish Academy of Sciences, Powstańców Warszawy 55, Sopot 81-712, Poland; Institute of Marine Research, PO Box 1870, Nordnes, Bergen 5817, Norway; Department of Arctic and Marine Biology, Faculty of Biosciences, Fisheries and Economics, UiT The Arctic University of Norway, Framstredet 39, Tromsø 9019, Norway; Department for Arctic Biology, The University Centre in Svalbard, PB156, Longyearbyen 9105, Norway; Faroe Marine Research Institute, PO Box 305 Nóatún 1 FO-110 Tórshavn, Faroe Islands; Alfred Wegener Institute Helmholtz Centre for Polar and Marine Research, Am Handelshafen 12, Bremerhaven 27570, Germany; AZTI, Marine Research, Basque Research and Technology Alliance (BRTA), Herrera Kaia, Portualdea z/g, Pasaia 20110, Gipuzkoa, Spain; IKERBASQUE—Basque Foundation for Science, Plaza Euskadi 548009 Bilbao, Bizkaia, Spain; Institute of Oceanology Polish Academy of Sciences, Powstańców Warszawy 55, Sopot 81-712, Poland; Centre for Ocean Life, National Institute of Aquatic Resources, Technical University of Denmark, Henrik Dams Allé, Building 202,2800 Kongens Lyngby, Denmark; Department of Ecoscience, Aarhus University, Frederiksborgvej 399, Roskilde 4000, Denmark; Alfred Wegener Institute Helmholtz Centre for Polar and Marine Research, Am Handelshafen 12, Bremerhaven 27570, Germany; Institute for Chemistry and Biology of the Marine Environment, Carl von Ossietzky University of Oldenburg, Carl-von-Ossietzky-Straße 9-11, Oldenburg 26111, Germany; Helmholtz Institute for Functional Marine Biodiversity at the University of Oldenburg (HIFMB), Ammerländer Heerstraße 231, Oldenburg 26129, Germany; Marine and Freshwater Research Institute, Fornubúðum 5220 Hafnarfjörður, Iceland; Institute of Oceanology Polish Academy of Sciences, Powstańców Warszawy 55, Sopot 81-712, Poland; Department of Arctic and Marine Biology, Faculty of Biosciences, Fisheries and Economics, UiT The Arctic University of Norway, Framstredet 39, Tromsø 9019, Norway

**Keywords:** zooplankton dynamics, habitat selection, environmental variables, North Atlantic, generalized additive model (GAM)

## Abstract

*Calanus finmarchicus* is an important, extensively studied zooplankton species in the North Atlantic. Many studies have explored its abundance and life cycle, but basin-wide relationships between its vertical distribution and environment during the feeding season remain poorly known. We conducted a meta-analysis of stage-specific vertical distribution and its relationships with environmental variables (temperature, salinity, irradiance, chlorophyll-*a*) in the epipelagic layer (0–200 m) of the North Atlantic during spring and summer (21 March to 21 September). Fitting a GAM model, we analyzed data from 47 years (1971–2018) with the aim to discern common, stage-specific responses to environment across the area. Highest abundances were observed in the upper 50 m in spring (at 5°C) and summer (at 7.5°C). The timing of the phytoplankton bloom emerged as a key driver determining vertical distribution, with all stages found shallower during the seasonal surface Chl.-*a* maximum. Contrary to reports of mismatch with global warming, the data indicated a region-wide match of spring bloom and *Calanus*. In the coldest areas of its habitat (< 1°C), the copepods stayed closer to surface, potentially to fulfill development, while in warmest areas (>10°C), early stages stayed deeper likely to avoid warm surface waters.

## INTRODUCTION

Zooplankton species have been studied for many centuries for reasons such as their pivotal role in the marine food web, their vertical migration, their key role in the oceanic carbon cycle, and for discovering and understanding the structure and biological dynamics of natural marine ecosystems (Russell, 1927; Darwin, 1933; Cushing, 1951; Steinberg and Landry, 2017). In the North Atlantic, the calanoid copepod *Calanus finmarchicus* has a key role in the zooplankton community due to its high abundance and biomass (Pershing and Stamieszkin, 2020). *C. finmarchicus* is one of the most studied zooplankton species and is of great importance for the development of many fish species, including some of commercial interest, such as Atlantic cod (*Gadus morhua*), herring (*Clupea harengus*), mackerel (*Scomber scombrus*), blue whiting (*Micromesistius poutassou*) and a variety of mesopelagic fish (Beaugrand *et al.,* 2003; Langøy *et al.,* 2012; Jacobsen *et al.,* 2020; Knutsen *et al.,* 2023).

*C. finmarchicus* performs extended ontogenetic seasonal vertical migrations: in late winter and spring copepodid stages ascend from overwintering depths, molt into adults and reproduce. The hatched nauplii develop in the upper water column growing on food available mainly from the spring phytoplankton bloom. After the six nauplii stages (NI–NVI), they progress through five copepodid stages (CI–CV), and in late summer and autumn CIV and especially CV copepodites descend to depths for overwintering again (Gislason and Astthorsson, 2000). Different copepodid stages have differential food demands, hence the copepodid stage and their growth and survival strategies influence their vertical distribution in the water column throughout the growing season (Vidal, 1980).

The habitat preference of *C. finmarchicus* is shaped by factors such as phytoplankton bloom dynamics, temperature variations, predators and their role individually or through combined relationships (Hirche *et al.,* 1997; Gaard *et al.,* 2008; Broms *et al.,* 2009; Basedow *et al.,* 2010; Lindegren *et al.,* 2020). *C. finmarchicus* shows high plasticity in life history traits with varying environmental conditions across its distributional range. This includes timing of reproduction and development time, number of generations per year and timing of seasonal descent to overwintering depth (Durbin *et al.,* 2000; Broms and Melle, 2007; Head *et al.,* 2013; Kristiansen *et al.,* 2021). Availability of phytoplankton in particular plays a key role in the copepod’s life cycle, and its adaptation to highly variable environments (Hirche *et al.,* 1997). In the Norwegian Sea, variability in the time and magnitude of *C. finmarchicus* spawning are driven by variations in hydrography and phytoplankton phenology, and in the Western North Atlantic food availability has been suggested as the main driver of short summer hibernation event (oversummering) observed in the species (Niehoff *et al.,* 2000; Saumweber and Durbin, 2006; Maps *et al.,* 2012).

While ocean circulation is mainly responsible for the horizontal distributions of planktonic species, ocean environmental conditions, such as temperature, food availability or the intensity of subsurface irradiance affect the vertical distributions of zooplankton (Unstad and Tande, 1991; Hobbs *et al.,* 2021; Omand *et al.,* 2021; Bandara *et al.,* 2021b). These abiotic and biotic factors also play a key role in governing the ontogenetic vertical habitat selection of *C. finmarchicus* (Kvile *et al.,* 2022). Although there are numerous observations of relationships between seasonal vertical distribution of *C. finmarchicus* and environmental characteristics, they do not provide a coherent picture throughout the North Atlantic. Most studies are performed in the context of separate projects and research programs and hence focus on specific areas of the North Atlantic or adjacent northern seas (Irigoien, 2000; Gaard *et al.,* 2008; Broms *et al.,* 2009; Lindegren *et al.,* 2020; Kaiser *et al.,* 2021). In contrast, meta-analyses that integrate all the above information may provide new insights of ecological interactions, community and ecosystem dynamics. However, reviews and meta-analyses of the data on this species predominantly focus on the annual cycle, phenology and life-cycle strategies, which are now well-documented (Gislason *et al.,* 2000; Broms and Melle, 2007; Melle *et al.,* 2014; Kristiansen *et al.,* 2021; Bandara *et al.,* 2021a). To our knowledge, there have been no comprehensive studies to date summarizing and comparing large sets of data on the vertical distribution data on *C. finmarchicus* in the upper water column in the North Atlantic and adjacent northern seas.

In this study, we aim to disentangle the stage-specific depth distribution of *C. finmarchicus* in relation to environmental variables (temperature, salinity, chlorophyll *a* (Chl.-*a*) and irradiance). We investigate this by performing a meta-analysis of the vertical distribution of *C. finmarchicus* in the North Atlantic and adjacent northern seas. We limited our vertical spatial focus on the upper pelagial (<200 m) and the temporal scope to the feeding season of *C. finmarchicus*, i.e. spring and summer between 1971 and 2018. The analyses were performed both for *C. finmarchicus* as a species, represented by all recorded life stages, and for individual life stages separately.

## METHODS

**Fig. 1:**
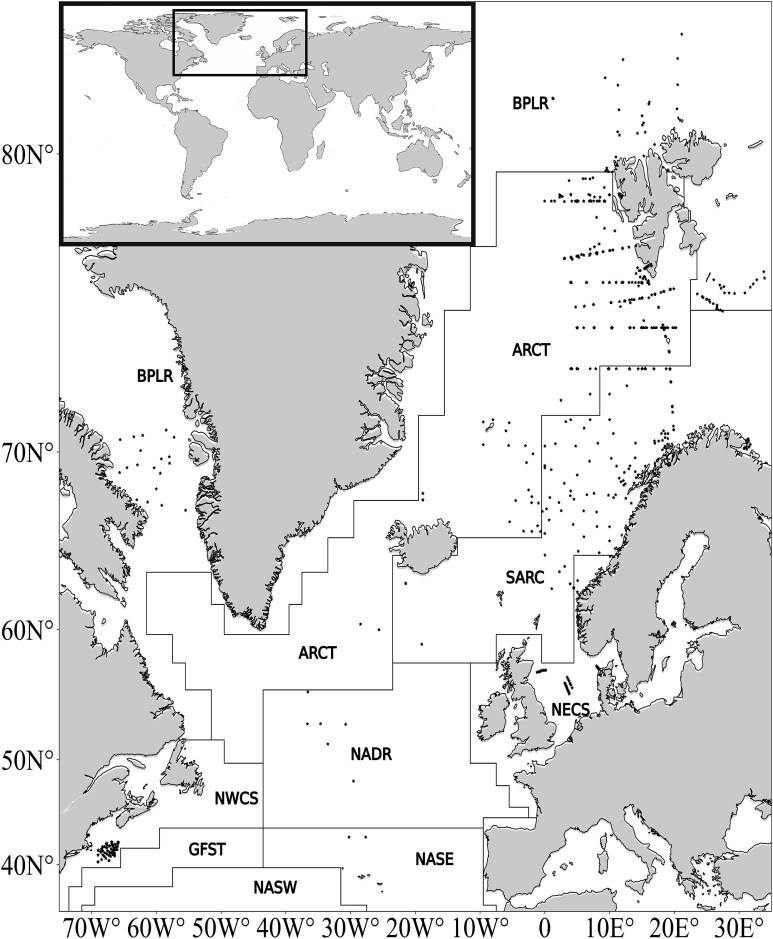
Station location (black stars) and habitat partition with the boundaries of the Longhurst’s provinces (Longhurst *et al.*, 1995; black lines). BPLR: Boreal Polar Province, ARCT: Atlantic Arctic Province, SARC: Atlantic Subarctic Province, NECS: NE Atlantic Shelves Province, NADR: N. Atlantic Drift Province, NWCS: NW Atlantic Shelves Province, GFST: Gulf Stream Province, NASE: N. Atlantic Subtropical Gyral Province (East), NASW: N. Atlantic Subtropical Gyral Province (West).

We compiled an extensive dataset comprising 1232 vertical profiles of the upper water pelagial (ranging from 5 to 200 m) of the North Atlantic. This dataset encompasses information on *C. finmarchicus* abundance across developmental stages and includes an array of environmental variables (temperature, salinity, Chl.-*a*, fluorescence and irradiance). The dataset includes observations from the North Atlantic basin and adjacent northern seas between 40°N to 82°N and 71°W to 34°E over five decades, spanning from 1971 to 2018 (Fig. 1). An overview of the data sources and locations of the sampling stations are described in Table I. Stations are located at selected marine regions according to Longhurst’s marine boundaries classification of the world’s oceans into provinces, based on physical forcing, which plays a dominant role in regulating phytoplankton distribution and life patterns (Longhurst *et al.,* 1995). To test our hypotheses on vertical distribution, we employed a generalized additive model (GAM) approach, integrating both environmental variables (temperature, salinity, surface Chl.-*a*, irradiance, time difference between spring bloom and sampling date) along with categorical factors (net type, region, month and mesh size).

**Table I:** *Overview of metadata for* Calanus finmarchicus *abundances and environmental variables sampling stations in the North Atlantic compiled for this study*.

Citation	Survey region	Vessel	Month(s) and year survey	Research institute	Number profiles	Sampling gear	Mesh size	Depth intervals	CTD	Chl.-*a*	Irradiance	Copepodid stages
(Broms *et al.,* 2009)	ARCT, SARC, NECS	R.V. “G.O. Sars” and R.V. “Johan Hjort”	March to August (1995)	IMR	104	MOCNESS	180 μm	200–100–50–25–0 m	Yes^a^	No	Yes	CI–CII–CIII–CIV–CV–AF–AM
(Head *et al.,* 2013)	SARC	R.V. “G.O. Sars” and R.V. “Johan Hjort”	July and October (1997)	IMR	6	MOCNESS	180 μm	100–50–20 m	Yes^a^	No	Yes	CI–CII–CIII–CIV–CV–AF–AM
(Trudnowska *et al.,* 2020b)	ARCT	RV “Oceania”	August (2016)	IOPAN	16	WP-2	60 μm	Bottom-50–0 m	yes	Yes (fluorometer)	no	CI–CII–CIII–CIV–CV–AF–AM
(Trudnowska *et al.,* 2020c)	ARCT/SARC	RV “Oceania”	June, July and August (2014, 2015)	IOPAN	18	MultiNet WP-2	180 μm 60 μm	Bottom-150–100–50–25–0 m bottom-100–50–0 m	Yes	Yes (fluorometer)	Yes	CI–CII–CIII–CIV–CV–AF–AM
(Trudnowska *et al.,* 2020a)	ARCT	RV “Oceania”	July (2018)	IOPAN	4	WP-2	180 μm	200–0 m/70–0 m/170–0 m	Yes	Yes	No	CI–CII–CIII–CIV–CV–AF–AM
(Trudnowska *et al.,* 2015)	ARCT	RV “Oceania”	July and August (2009, 2010)	IOPAN	12	MPS	180 μm	50–40–30–20–10–0 m		Yes (fluorometer)	no	CI–CII–CIII–CIV–CV–AF
(Trudnowska *et al.,* 2014)	ARCT	RV “Oceania”	July and August (2012)	IOPAN	4	MPS	180 μm	Bottom-50 ~ 40 -0 m	yes	Yes (fluorometer)	yes	CI–CII–CIII–CIV–CV–AF–AM
	ARCT/SARC	Non-specified	June and July (2001–2015)	IOPAN	385	Non-specified	Non-specified	Non-specified	Yes	No	No	CI–CII–CIII–CIV–CV–AF–AM
	ARCT/SARC	Non-specified	May and June (1990–2013)	MRI	67	WP-2	200 μm	50–0 m	Yes^b^	Yes	Incomplete	CI–CII–CIII–CIV–CV–AF–AM
(Daase *et al.,* 2008)	ARCT/BPLR	RV “Jan Mayen”	September 2002	UNIS	6	MultiNet	180 μm	300–200–150–100–50–0 m	Yes	Yes (fluorometer)	Incomplete	CI–CII–CIII–CIV–CV–AF–AM
(Daase and Eiane, 2007)	ARCT/BPLR	RV “Jan Mayen”	August and September (2003, 2004)	UNIS	38	MultiNet WP-2	180 μm	Non-uniform	Yes	Yes (fluorometer)	incomplete	CI–CII–CIII–CIV–CV–AF–AM
(Gaard *et al.,* 2008)	ARCT/NADR/NASE	RV “G.O. Sars”	June and July 2004	FAMRI	11	MPS	180 μm	200–100–0 m	Yes^a^	No	Yes	(CI–CIII)–CIV–CV–AF–AM
(Durbin and Casas, 2012)	NWCS	RV “Albatross IV”, RV “Endeavor” and RV “Oceanus”	March, April, May, June and July (1995–1999)	URI	100	MOCNESS	335 μm	Non-uniform	Yes	No	Yes	CI–CII–CIII–CIV–CV–AF–AM
(Kaiser *et al.,* 2021)	ARCT	RV “Polarstern”	July 2017	BreMarE	4	MultiNet Midi	150 μm	300–200–100–50–10–0 m	Yes	No	Yes	(CI–CIII)^c^–CIV^b^–CV–AF–AM
(Balazy *et al.,* 2019)	ARCT	RV “Oceania”	July and August (2015, 2016)	IOPAN	39	WP-2	180 μm^e^	50–0 m	Yes^c^	No	Incomplete	CI–CII–CIII–CIV–CV–AF–AM
(Lindegren *et al.,* 2020)	NECS	RV “Dana”	August and September (2002, 2003, 2005, 2007)	DTU Aqua	38	Plankton net fitted to a submersible pump (Homa, H-500)	200 μm	Bottom-50–40–30–20–10–0 m	yes	Yes (fluorometer)	incomplete	CV^d^- AF–AM
(Bucklin and Manning, 2007)	NWCS	Non-specified	April (2002) to March (2003)	OPAL	100	MOCNESS	150 μm	Non-uniform	Yes	No	No	*C. finmarchicus* or *C. finmarchicus* younger than C6
(Kjellerup *et al.,* 2015)	BPLR	RV “Bjarni Sæmundsson”	September (2009)	AU-BIOS	24	MultiNet Midi Bongo net	45 μm 335 μm	500 (or bottom)-200–150–100–50–0 m/0–50 m and 0–200 m	Yes	Yes (fluorometer)	Yes	CI/CII^e^–CIII–CIV–CV–CVI
(Häfker *et al.,* 2018)	NECS	RV “Calanus”	March, May, June and August (2015–2016)	AWI	6	WP-2	200 μm	5–50 m and 50–45 m	Yes	Yes (fluorometer)	No	CI–CII–CIII–CIV–CV–AF–AM
(Irigoien, 2000)	SARC	St. India	March to October (1971–1975)	PML	108	LHPR	280 μm	Every 10 m from 500 m depth to the surface	yes	Yes (from water samples in mg.m-3)	no	CI–CII–CIII–CIV–CV–CVI
(Zhou *et al.,* 2009)	SARC	Non-specified	June (2000)	UiT	2	MOCNESS	180 μm	Non-uniform	Yes	Yes (fluorometer)	No	CI–CII–CIII–CIV–CV–CM–CF
(Basedow *et al.,* 2010)	SARC	RV “Jan Mayen”	May and June (2002)	UiT	27	MOCNESS	180 μm	0–20–40–60–80–100–120 m	Yes	Yes (fluorometer)	Yes	CI–CII–CIII–CIV–CV–CM–CF
	SARC	RV “Johan Ruud”	June (2008)	UiT	2	Multinet	Non specified	0–15–30–50–75–100 m	Yes	Yes (fluorometer)	Yes	CI–CII–CIII–CIV–CV–CM–CF
	BPLR/SARC	RV “Jan Mayen”	April and May (2008)	UiT	15	Multinet	Non-specified	20–40–60-100	yes	Yes (fluorometer)	Yes	CI–CII–CIII–CIV–CV–CM–CF
	SARC	RV “Johan Ruud”	May (2009)	UiT	4	Multinet	Non specified	Non-uniform	Yes	No	Yes	CI–CII–CIII–CIV–CV–CM–CF
	SARC	Non-specified	June (2018)	UiT	18	MPS	180 μm	Non-uniform	Yes	Yes (fluorometer)	Yes	CI–CII–CIII–CIV–CV–CM–CF
	SARC	Non-specified	April and May (2017)	UiT	9	MPS	180 μm	Non-uniform	Yes	Yes (fluorometer)	Yes	CI–CII–CIII–CIV–CV–CM–CF
(Basedow *et al.,* 2018)	BPLR/ARCT	RV “Helmer Hanssen”	May and August (2014)	UiT	14	MultiNet Midi	180 μm	Non-uniform	Yes	Yes (fluorometer)	Yes	CI–CII–CIII–CIV–CV–CM–CF
	BPLR	RV “Jan Mayen”	August (2007)	UiT	13	Non-specified	Non-specified	100–75–50–25–0 m	Yes	Yes (fluorometer)	Yes	CI–CII–CIII–CIV–CV–CM–CF

IMR: Institute of Marine Research, IOPAN: Institute of Oceanology Polish Academy of Sciences, UNIS: The university center in Svalbard, FAMRI: The Faroese Marine Research Institute, URI: The University of Rhode Islands, BreMarE: Bremen Marine Ecology—Centre for Research & Education, DTU: Technical University of Denmark, OPAL: Ocean Process Analysis Laboratory, University of New Hampshire, AWI: Alfred Wegener Institute for Polar and Marine Research, PML: Plymouth Marine Laboratory, AU-BIOS: Aarhus University, Department of Bioscience, UiT: The Arctic University of Norway, MPS: Multi Plankton Sampler, LHPR: Longhurst-Hardy Plankton Recorders, CI: Copepodite stage 1, CII: Copepodite stage 2, CIII: Copepodite stage 3, CIV: Copepodite stage 4, CV: Copepodite stage 5.

^a^CTD from ICES repository.

^b^ *C. finmarchicus*/*glacialis*.

^c^Salinity missing in some stations.

^d^Medium stage includes only CV.

^e^ *Calanus* spp.

^f^CTD averaged 50–0 m.

^g^station K2 samples (60 um).

### Environmental data

Initially, the environmental variables included were sea temperature and salinity, recorded by conductivity–temperature–depth (CTD) profilers used at stations where the zooplankton nets were deployed. Fluorescence, and Chl.-*a* biomass coming from sensors and water samples were excluded due to poor availability (13% of the profiles). Hence, surface Chl.-*a* biomass was extracted from satellite data (29% of the profiles). Irradiance was computed using the location, the sampling time, and the cloud cover (see below). Data related to the sampling process are listed in Table I. Temperature and salinity were averaged across 5-m depth bins, with the exemption of data from Iceland where CTD measurements were averaged within the depth interval of 0 to 50 m.

### Irradiance intensity estimation

The irradiance was estimated as previously described by Bandara *et al.* (2018) derived from a global horizontal irradiance model of Robledo and Soler (2000), which assumes clear sky conditions. To account for cloud attenuation of the estimated clear-sky irradiance, the total cloud cover (1979–2021) from the ERA5 reanalysis dataset were used (Hersbach *et al.,* 2023). The temporal resolution of cloud cover data used was hourly. Cloud-attenuated sea surface irradiance was calculated following Bandara *et al.* (2022). Further, subsurface irradiance attenuation equations were used to estimate the irradiance at each 5-m bins. Irradiance could be computed for ~ 40% of the dataset due to missing information (time or cloud cover) for the rest of profiles (Wetzel, 1983).

### Surface chlorophyll concentration and bloom development

Surface Chl.-*a* data were accessed from the EU Copernicus Marine Service Information (https://doi.org/10.48670/moi-00281). The Global Ocean Satellite Observations, ACRI-ST company (Sophia Antipolis, France) provides Bio-Geo-Chemical products (daily and interpolated) based on the Global Ocean Color (Copernicus-GlobColour). In this study, we used surface Chl.-*a* concentration with a 4 km spatial resolution from 1997 to the present. The maximum of surface Chl.-*a* in spring or autumn is an indicator of the phytoplankton bloom which has been suggested to influence C*alanus* spp*.* behavior (Broms and Melle, 2007). Therefore, from surface Chl.-*a* concentration we estimated the time gap, in number of days, between the surface Chl.-*a* maximum (surface bloom) and the sampling date (Δday_CHLmax_) at each sampling location. A 180-day window around the sampling date at each sampling location was used to identify the surface Chl.-*a* maximum. The large time span was used to be able to identify either the spring or a subsequent (autumn) phytoplankton bloom.

### *C. finmarchicus* net sampling data

*C. finmarchicus* abundance data were collected using different net sampling techniques, including WP-2 (200 180 and 60 μm), Multi Plankton Sampler (MPS; 180 μm), Mocness (335, 180 and 150 μm), MultiNet (180, 150, 53 and 45 μm), Plankton net (200 μm), Bongo net (335 μm) or Longhurst Hardy Plankton Recorder (LHPR; 280 μm; Table I). These devices provide species abundance throughout the water column, but due to the diversity of mesh sizes the selectivity could vary between stages. This diversity in sampling methods introduces several complexities. In this study, we assume that early stages may be under sampled using larger mesh sizes, while adults could be under sampled using smaller mesh sizes (Hopcroft *et al.,* 2005).

To allow statistical analysis and inter-comparison with environmental variables, for each developmental stage and each profile abundance data were estimated for every 5-m depth bin. First, 1-m bins were associated with their corresponding abundance in ind m^−3^ from the original sampling resolution (Table I). For this it was assumed that copepods were equally distributed in the sampled layer, i.e. if e.g. the sampled abundance in a 20–0 m layer was 10 ind m^−3^, it was assumed that each 1-m bin between 0 and 20 m had an abundance of 10 ind m^−3^. Then, 5-m bins of abundance were derived by calculating the mode of that bin based on the 1-m bins. In contrast to a mean, the mode is the most common set of numbers. The resulting dataset contained 5-m bins, between 5 and 200 m depth. We acknowledge that differences in the resolution of the original sampling will influence the accuracy of this method; however, this is an assumption we must accept within the scope of this study.

**Fig. 2:**
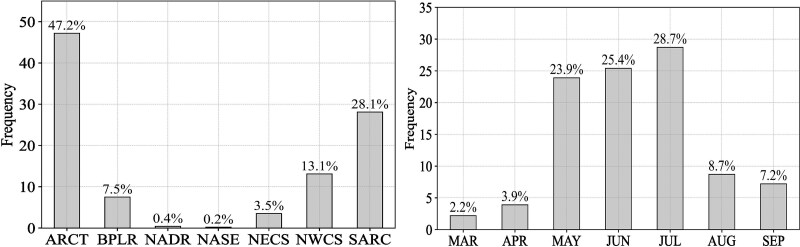
Frequency plot of the percentage of vertical profiles within regions of the North Atlantic (left) and across spring and summer months (right).

In compiling data for the present study, we focused on spring and summer (21 March–21 September; Fig. 2), aligning with the period when *C. finmarchicus* ascends to the upper water column after the overwintering period, reproduces and grows. The different stages were identified according to the study protocols of the corresponding research projects (Table I). It is now clear that prosome length is not a valuable criterion for distinguishing between *C. glacialis* and *C. finmarchicus* (Choquet *et al.,* 2018). However, this approach was wide-spread earlier, before genetic analyses revealed the overlap in size between the two species. This potential misidentification during that era adds another layer of complexity and potential bias to the dataset, emphasizing the importance of cautious interpretation when considering historical data in regions where species discrimination may have been challenging. In the present dataset, not all studies identified younger developmental stages CI to CIII, therefore our dataset includes copepodid [C] early stages (ES; CI–CIII), stage CIV, stage CV and adults, encompassing both adult females (AF) and adult males (AM).

### Data preparation for statistical analysis

Before fitting the statistical model, we pre-processed the data as follows. First, for each vertical profile the vertical distribution of *C. finmarchicus* was estimated by computing weighted mean depth (WMD_m_) using the method described by (Manly, 1977) as:


$${\left({WMD}_m\right)}_{d,t,x,y}=\frac{1}{2}\sum_{j=1}^n\frac{{\left({f}_j\right)}_{d,t,x,y}{d}_j{z}_j}{O}$$

where *n* is the number of depth intervals, *d_j_* = lower sample—upper sample depth [m] of sample interval *j, z_j_* is the mid strata [m] of sample interval *j, f_j_* is the density of individuals [m^−3^] observed in depth interval *j,* and *O* corresponds to the area under the frequency curve (i.e. the estimated surface integrated abundance):


$$ O=\frac{1}{2}\sum_{j=1}^n{d}_j\times{f}_j $$

Second, we averaged over the vertical profiles (0–200 m) to obtain the mean temperature, the mean salinity, and the mean irradiance, for each associated WMDs. Third, the abundances were also averaged across different developmental stages (early stages (CI–CIII), CIV, CV, AF and AM) since WMD was stage-specific for each vertical profile. Finally, surface Chl.-*a* biomass and Δday_CHLmax_ were associated to each WMD and the categorical variables month, marine region, sampling time, mesh size and sampling device (MOCNESS, MPS, MultiNet, WP2, submersible pump (Homa, H-500) or LHPR) were also included. The abundances of *C. finmarchicus* were transformed using a *log_10_*(x + 1) function to reduce skewness and to ensure that residuals were normally distributed (Crawley, 2002).

### Modelling the WMD of *C. finmarchicus*

To understand the relationship between *C. finmarchicus* WMD and abiotic and biotic environmental variables, five different models were fitted, each tailored to a specific stage, early stages (CI–CIII; *n* = 328), CIV (*n* = 311), CV (*n* = 309), AF (*n* = 265) and AM (*n* = 150). To account for potential non-linear relationships between the response variable (WMDs) and the predictor variables (environmental variables) we used GAM. All statistical model analyses were performed using R Statistical Software (v4.2.2; R Core Team, 2021). Model selection was done using the *mgcv* package in RStudio (v1.8–34; Wood, 2011). Model selection included the backward and elimination method for the detection of predictors to be retained in the final models. In this method, we began with all predictors, then removed the least significant ones until the most simple model based on Akaike’s information criterion was selected (AIC; Akaike, 1974). The models with smaller AIC and with the predictors statistically significant at the 10% level was selected. The spline smoother function (s) was constrained to three knots (k = 3) to allow for potential nonlinearities but restrict flexibility during model fitting. The ultimate selection was based on AIC comparison and predictors significance (selected models are highlighted in gray; Table A1). Final models adjusted *k* for each covariate to minimize AIC (Table II). As part of the pre-analysis data exploration, scatter plots were produced to examine relationships between predictor variables (salinity, temperature, abundance, irradiance, sampling time, surface Chl.-*a*, number of days between the maximum of surface Chl.-*a* and sampling day). The objective was to identify any potential collinearities that could affect subsequent analyses. After examination, no significant collinearities were detected among the predictor variables. Also, residual inspection identified outliers for surface Chl.-*a* values exceeding 2 mg/m^3^, and these outliers were subsequently removed in the final model fitting process. Marine region, month, sampling device and mesh size were considered as categorical factor and included as a random intercept in all the models.

**Table II:** *Selected models use to fit dataset, AIC scores and R2 values for selected model*.

Model selected for each stage	AIC	R^2^	Deviance explained
WMD_ES ~ s(Temp***) + s(sal**) + s(time*) + Marine Region+Month+s(Net_ES) + s(Mesh_ES) + s(Net_ES, Mesh_ES**)	2 873	0.57	60.7%
WMD_CIV ~ s(Temp***) + s(sal***) + s(Chlsat*) + s(time**) + s(abun ●) + Marine Region+Month+s(Net_ES) + s(Mesh_ES) + s(Net_ES, Mesh_ES***)	3 007	0.56	60%
WMD_CV ~ s (Temp●) + s(sal***) + s(Chlsat ●) + s(time*) + Marine Region+Month + s(Net_ES***) + s(Mesh_ES***)	3 030	0.55	58.6%
WMD_AF ~ s(Temp**) + s(sal***) + s(time***) + Marine Region+Month +s(Net_AF) + s(Mesh_AF) + s(Net_AF, Mesh_AF***)	2 503	0.66	68.8%
WMD_AM~s(Temp*) + s(sal***) + s(Chlsat***) + s(abun*) + Marine Region+Month+ s(Net_AM, Mesh_AM***)	1 460	0.55	62.2%

The vertical position of *Calanus finmarchicus* was modeled depending on environmental factors, based on data collected in the North Atlantic during spring and summer from 1970 to 2019. The first column shows the selected model that fitted the data best. Statistical models were fitted for the different development stages: early stages (ES), CIV, CV, AF and AM where the covariates are marked with their significant codes: “***” 0.001 “**” 0.01 “*” 0.05 “^●^” 0.1. The second columns show the value of AIC for each model and the third column the adjusted coefficient of determination (R^2^ adjusted). The last column shows the deviance explained by each model.

A significant effect in the GAMs is detected when the response variable (WMD) deviates from zero. Each curve fitted to WMD against a predictor (temperature, salinity, etc.) shows the partial effect that the predictor had on the WMD. The final models showed a generally good fit to the data, with R^2^ values spanning from 0.55 to 0.68 and the deviance explaining percentages from 58.6% to 68.8%. Overall, the models performed well and captured the key patterns in the data across the different developmental stages (Table II). The residual plots from the model were normally distributed and despite the minor deviations thus suggest a good fit of the model (Fig. A1). The best GAM models fitted for each developmental stage are detailed in Table II. Environmental variables were included in the models only if they were found to have a statistically significant effect on WMD. All models included the categorical variables of marine region and month to account for potential spatial and temporal variations that could influence the vertical distributions. Additionally, details of the sampling devices, including net type and mesh size, were found to have a significant effect on WMD for all developmental stages and were included in the models, either independently or as interaction terms, to achieve the best model fit (Table II). Based on emerging results from the GAM, we explored relationships to temperature and season with single-factor analyses of variance (ANOVAs) to highlight these responses.

## RESULTS

### Hydrography and chlorophyll-*a*

**Fig. 3:**
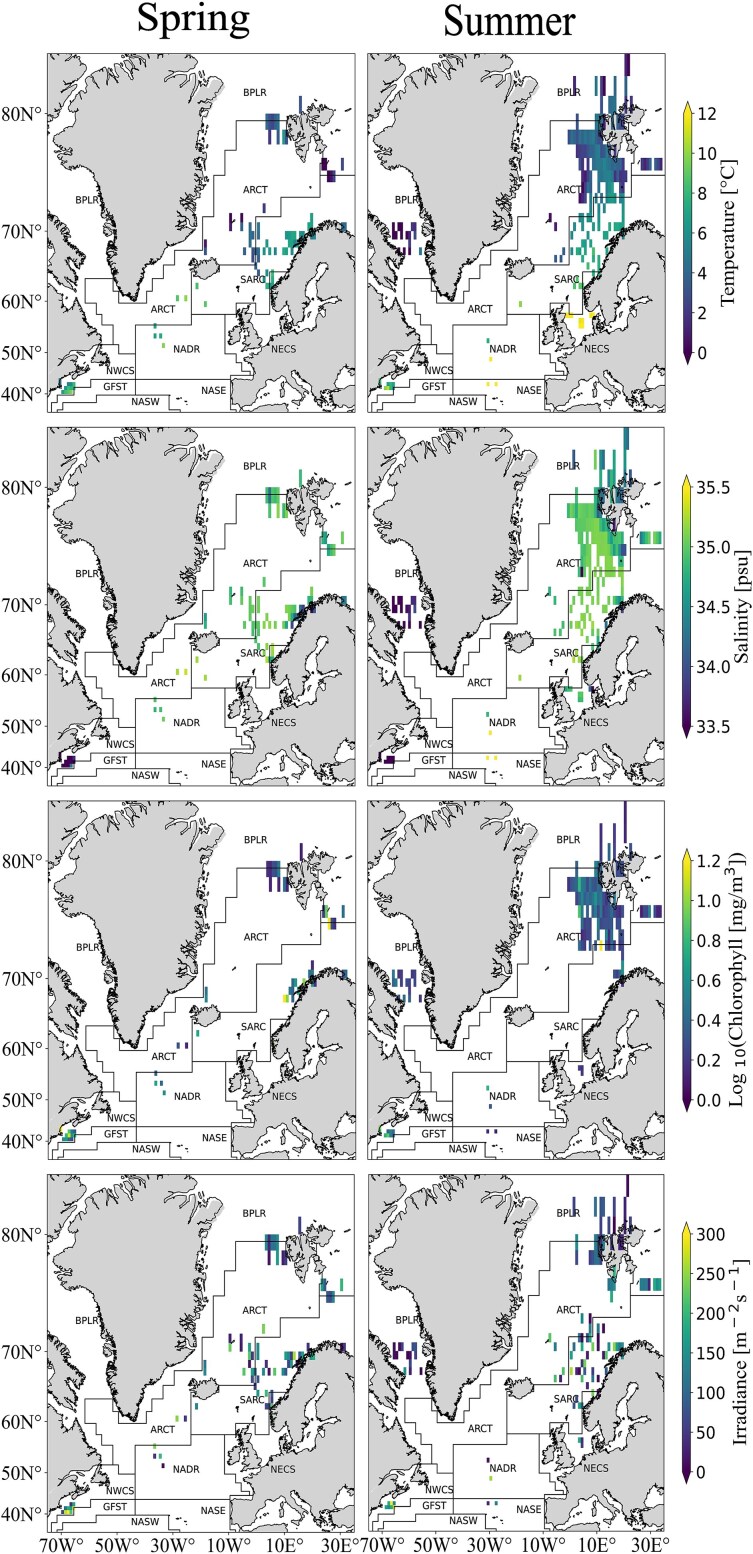
Distribution of environmental variables (temperature, salinity, surface Chl.-*a* and irradiance) of the vertical profiles across the marine regions where data were collected. Values shown are means for each vertical profile. Spring extends from 21 March to 20 June and summer from 21 June to 22 September.

Unsurprisingly, the lowest water temperatures in the data set from the upper pelagial (<200 m) were recorded in the northernmost region, and the highest in the southernmost region (Fig. 3). A clear temperature gradient from south to north was evident throughout the North Atlantic and adjacent northern seas. Furthermore, in all regions mean water column temperatures increased from spring to summer, between 0.07 (SARC region) and up to 4°C (NECS region). This trend was not found in the NASE region, where data were only recorded during summer (Fig. 3). Salinity values differed between regions, with the highest salinity being observed within the southernmost region and the lowest in the western North Atlantic along the North American coast. Variation from spring to summer was particularly pronounced in the northernmost area and in the North Sea, in these regions salinity increased by 0.7 psu from spring to summer (Fig. 3). Irradiance increased from spring to summer, with highest levels being recorded in the southern regions (Fig. 3). Apart from that, no regional trend was observed within the irradiance data. Surface Chl.-*a* concentrations varied significantly with sampling date but not related to season or region. High values occurred both during spring and summer sampling events, and within all regions (Fig. 3). Most data were collected during periods with expected timing of the phytoplankton bloom; however, no clear maxima of Chl.-*a* concentrations were observed during spring season. This lack of high values of surface Chl.-*a* despite the productive season may have been partly due to the timing of sampling, as 18% of the stations were sampled more than 50 days before or after the surface Chl.-*a* maximum (Δday_CHLmax_ > 50).

### Seasonal change of stage-specific *C. finmarchicus* abundance

**Fig. 4:**
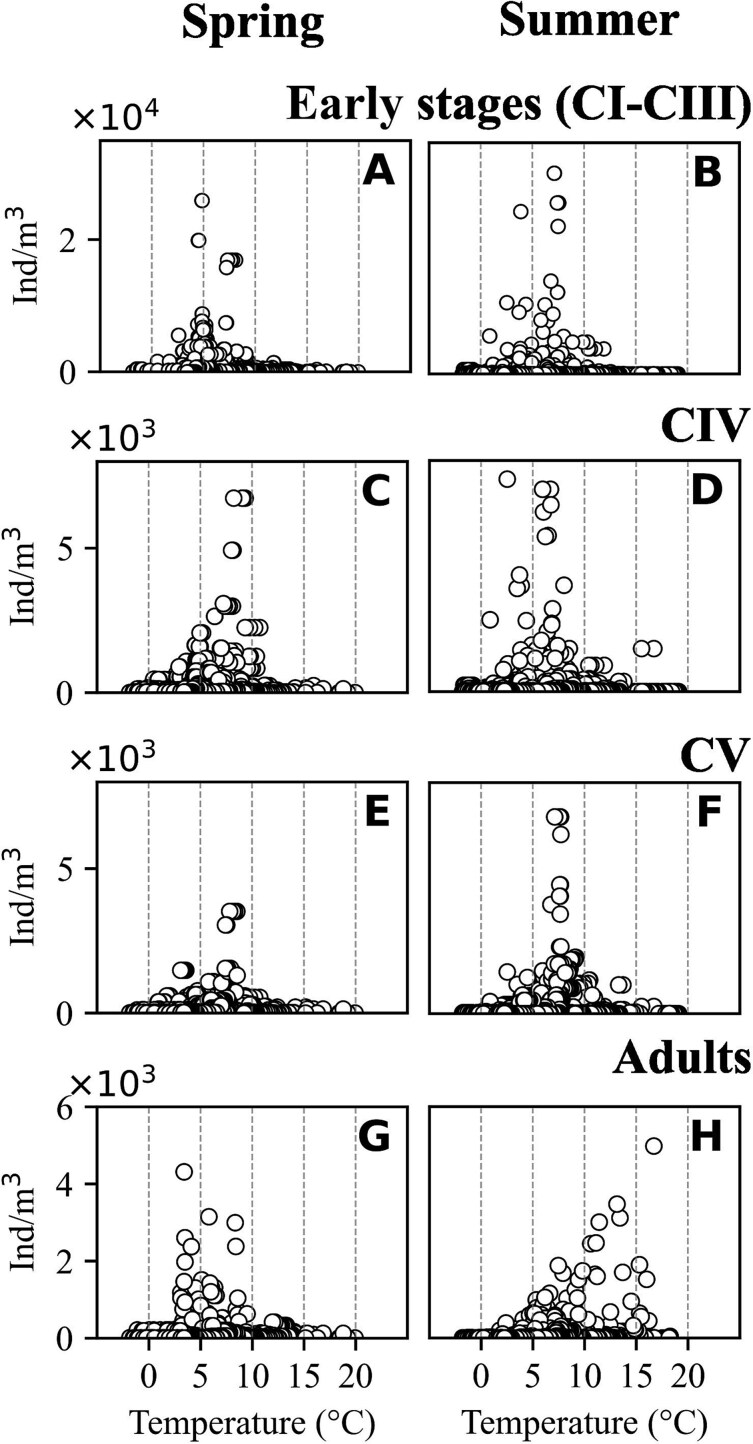
Stage-specific *Calanus finmarchicus* abundances (y-axis, ind m^−3^) in relation to temperature (x-axis, °C) in spring (left panels) and summer (right panels) in the North Atlantic. Based on averaged data from vertical profiles from all regions.

**Fig. 5:**
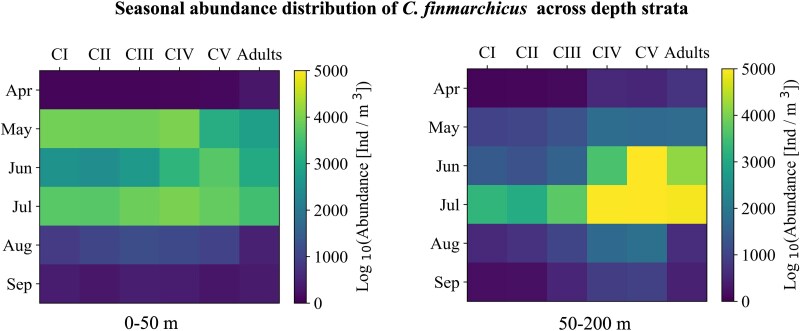
Seasonal variation in stage-specific *Calanus finmarchicus* abundance in the upper (0–50 m; left) and lower (50–200 m; right) part of the upper water column.

In spring peak abundances were observed at lower temperatures than in summer, this was true for all stages except from CIV (Fig. 4). In the data set, the early stages (CI–CIII) had the highest abundances, with their maximum abundance peaking at around 5°C in spring (Fig. 4a) and around 7.5°C in summer (Fig. 4b). For developmental stage CIV, the highest abundance in both summer and spring was observed at around 5–6°C (Fig. 4c and d). In summer the highest numbers of the CV developmental stage were recorded, with the highest concentrations observed at around 7°C (Fig. 4f). A shift toward higher temperatures in summer was also observed for the adults (AF and AM; Fig. 4g and h). The early developmental stages CI–CIII were mainly observed from May to July, with higher abundances in May and July compared to June (Fig. 5). In July, these stages occurred both in the upper 50 m and below, while in May and June they were observed almost exclusively in the upper 50 m. The older stages occurred mostly in the upper 50 m in May but were found in highest abundances below 50 m in June and July (Fig. 5).

### Stage-specific vertical distribution in relation to hydrography

**Fig. 6:**
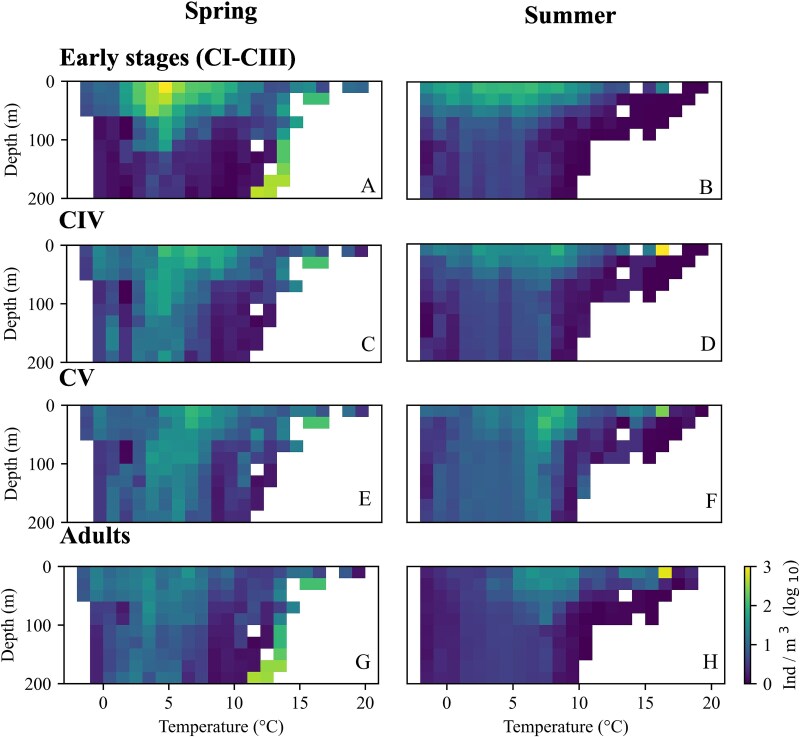
Vertical distribution of *Calanus finmarchicus* developmental stages averaged for sequential temperature values (x-axis). Based on 1232 vertical profiles in the North Atlantic. Left: spring (21 March to 20 June), right: summer (21 June to 22 September).

**Fig. 7:**
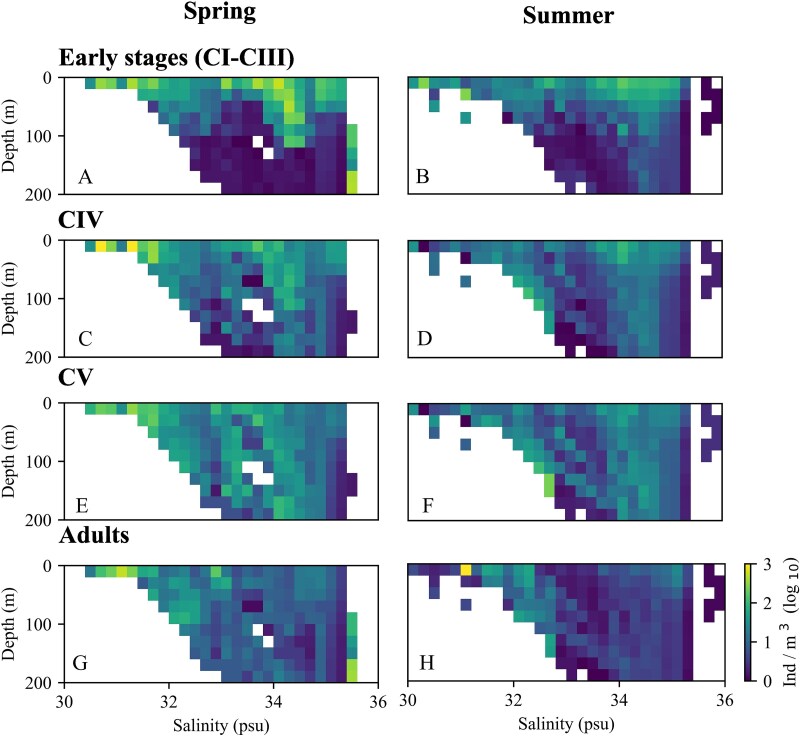
As Fig. 6, but for sequential salinity values.

The vertical distribution of *C. finmarchicus* developmental stages varied in relation to temperature and salinity (Figs 6 and 7). At most temperatures recorded, the peak abundances of all stages occurred in the upper 50 m, apart from one notable exception with the relatively high abundances of early stages and of adults at temperatures > 10°C below 100 m originating from one station in the NWCS region. During spring, early stages were highly abundant at 5°C and predominantly found in the upper 20 m. Overall, highest densities of all stages were observed within a temperature range of 3 to 8°C (Fig. 6). CVs were most abundant at 7.5°C in the upper 50 m in summer, however overall abundances tended to be lower in summer (Fig. 6). At temperatures above 10°C abundances tended to be very low in summer, with the exception of one profile in the NWCS region where relatively high abundances of all stages were observed at 17°C. The vertical distribution in relation to salinity was less homogeneous (Fig. 7) than the one in relation to temperature. Highest abundances of all developmental stages were observed at lower salinities in surface waters in spring, but relatively high abundances were also recorded in surface waters (early stages) and below 50 m (older stages incl. adults) at higher salinities. In summer, higher abundances of early stages tended to be observed at higher salinities, while relatively high abundances of older stages were observed both at lower and at higher salinity values. (Fig. 7).

### Stage-specific weighted mean depth in relation to environmental factors

**Fig. 8:**
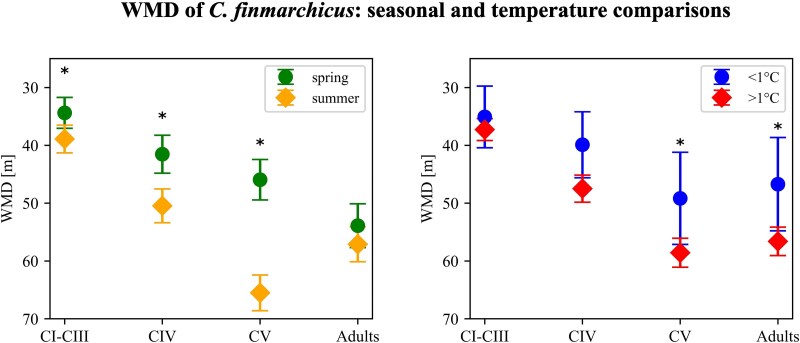
WMD of *Calanus finmarchicus* developmental stages in spring and summer (left) and at temperatures of < 1°C and > 1°C (right). Error bars show confidence intervals at 95% confidence level. Based on averaged WMD for profiles with matching characteristics. Significant differences are marked with *.

**Fig. 9:**
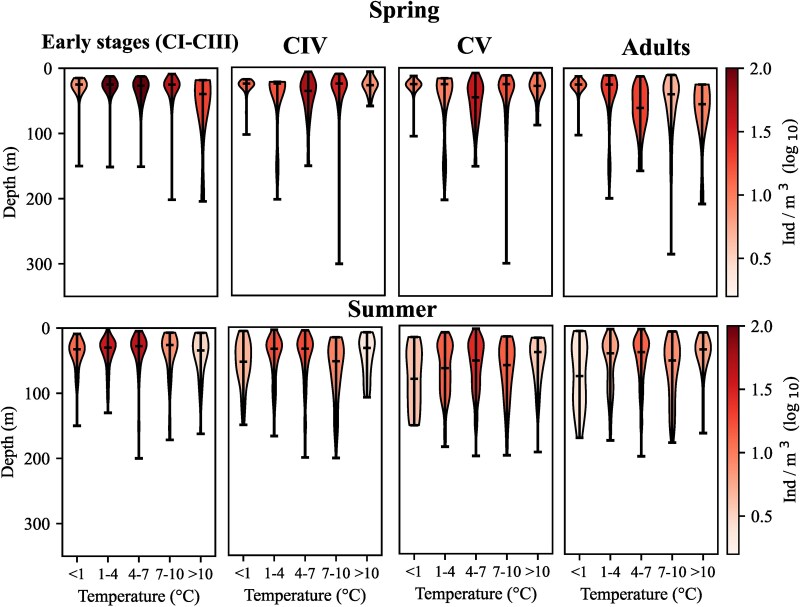
Violin plot representation of the WMD and mean abundances of *Calanus finmarchicus* stages by mean temperature intervals within the upper water column in spring and summer. The black/gray middle horizontal line denotes median values, while the upper and lower ones represent maximum and minimum respectively.

**Fig. 10:**
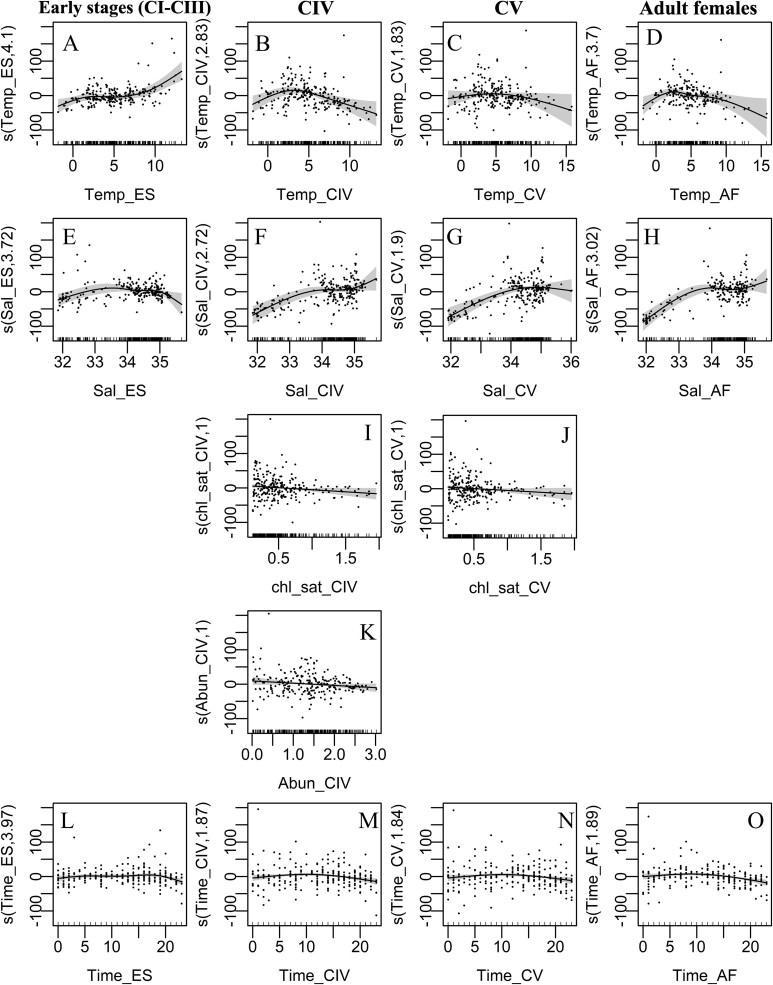
Effect of significant variables (temperature, salinity, surface chlorophyll-*a,* abundance and time) on the vertical distribution (weighted mean depth) of *Calanus finmarchicus* for each development stage (except AM included in appendix, Fig. A3). Partial effect (solid line), 95% confidence intervals (shadow area) and residuals (gray dots) are depicted as well. The model was built using *Calanus* distribution data obtained from samples collected in the North Atlantic in spring and summer using various methods (see Table I).

Based on the examined data set, the mean depth distribution of *C. finmarchicus* varied depending on the developmental stage (ANOVA, *P* < 0.001), with early stages occupying significantly shallower depths (WMD_ES_ = 37 m; σ = 30) than CIVs (WMD_CIV_ = 46 m; σ = 37), CVs (WMD_CV_ = 58 m; σ = 40) and adults (WMD_CA_ = 56 m; σ = 41) (Fig. 8). Furthermore, all stages were distributed shallower in spring than in summer (ANOVA, *P* < 0.05; Fig. 8). Temperature influenced the WMD of *C. finmarchicus* (Figs 8 and 9). To explore potential different response in habitats with and without ice, we compared vertical distributions at temperatures < 1°C and > 1°C respectively. The WMD of all stages was shallower at temperatures < 1°C than in warmer waters (Fig. 8). In spring, the WMD of all stages was centered within the upper 50 m at temperatures below 1°C (Fig. 9). In contrast, in summer CIV to adults tended to stay deeper at temperatures < 1°C (Fig. 9). The GAM analyses confirmed the significant effect of temperature on the WMD of all developmental stages (Table II). The WMD of early stages was shallower at temperatures below 1°C and deeper at temperatures above 10°C (Fig. 10a) and the WMD of CIV, CV and adults, was shallower in both colder and warmer waters, with the greatest depths observed around 5°C (Fig. 10b–d, Fig. A3a). Salinity was also found as a significant factor for all developmental stages (Table II). At salinities < ~ 32 psu and > ~ 35 psu, early stages showed a slightly shallower distribution than at intermediate salinity and CIV, CV and AF were distributed shallower at salinities < ~ 32 psu, and gradually deeper toward the average weight mean depth at ca. 34 psu. Finally, at higher salinities, CIV copepodites and AF were distributed slightly deeper (Fig. 10e, f and h).

Two other parameters, abundance and sampling time significantly improved the fit of the GAM and were found to influence the WMD of some developmental stages (Table II). A significant density-dependent effect on WMD was observed for CIV copepodites and AM, which were observed deeper in the water column at low *Calanus* densities (Fig. 10k, Fig. A3). Although time was a significant factor (Table II), the GAM results showed little to no variation of vertical distribution with time of the day (Fig. 10l–o).

**Fig. 11:**
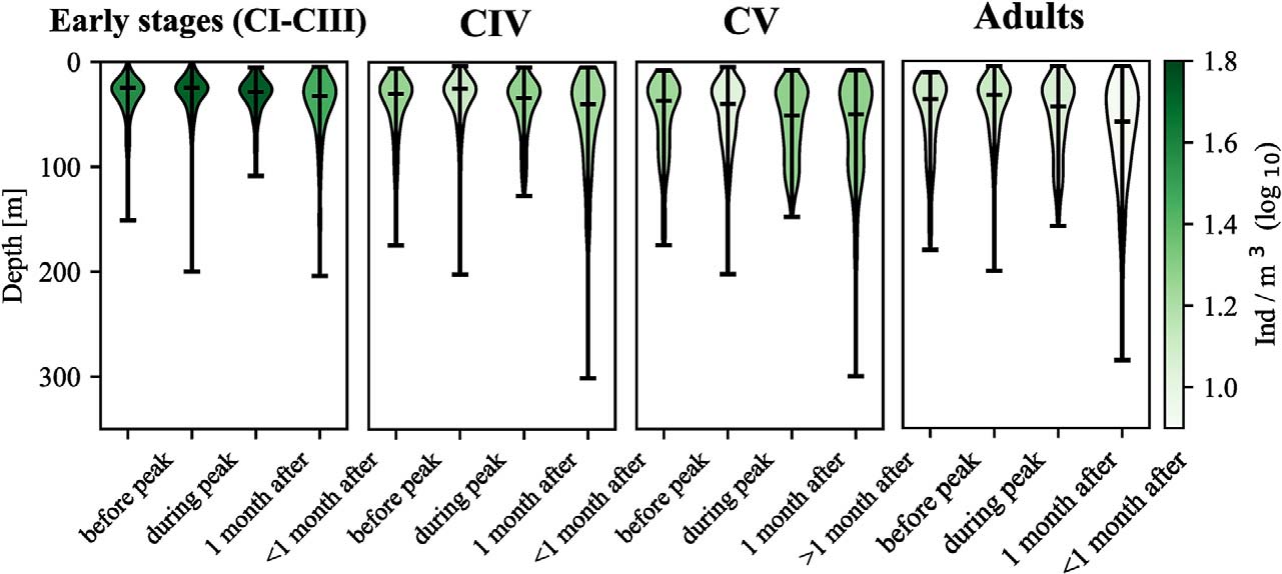
Violin plot representation of the WMD and mean abundances of *Calanus finmarchicus* stages before, during and after the chlorophyll-*a* peak of the year. Horizontal lines as in Fig. 9.

The effect of surface Chl.-*a* on WMD when fitting the GAM was only significant for CIV, CV (Fig. 10i and j) and AM (Fig. A3). CIV and CV copepodites were distributed slightly closer to the surface at Chl.-*a* concentrations > 1.5 mg m^−3^. Chl.-*a* smoothers showed a slightly shallower position in the water column of CIV, CV and adults males at higher surface concentrations (Fig. 10i and j, Fig. A3). AM stayed deep at low surface Chl.-*a* concentrations. Surface Chl.-*a* concentrations > 1.5 mg m^−3^ can indicate an ongoing phytoplankton bloom; we therefore analyzed the vertical distribution of copepods in relation to the timing of the phytoplankton bloom. To asses this relationship, we calculated the number of days between the sampling date and the peak of surface Chl.-*a* concentration grouping the results into four categories: before Chl.-*a* peak, during Chl.-*a* peak, within one month after the Chl.-*a* peak, and more than one month after the Chl.-*a* peak (Fig. 11). During the seasonal peak of surface Chl.-*a*, all developmental stages were centered in the upper 50 m of the water column. Particularly, early stages (CI–CIII) exhibited a significant tendency to position themselves shallower during the phytoplankton bloom period and for up to one month thereafter. Also, one month prior and one month after the seasonal Chl.-*a* peak, the WMD were within the upper 50 m for all stages apart from CV copepodites, which stayed slightly deeper in the water column one month after the bloom (Fig. 11). Mean abundances in the upper water column increased one month after the Chl.-*a* peak for early stages, CIV and CV. Abundance of adults was slightly higher during the bloom and their distribution in the water column showed a shallower depth range during the peak compared to after the peak (Fig. 11). However, the number of days between the sampling date and the seasonal peak did not significantly contribute to the GAM fit and was therefore excluded from the model for all developmental stages.

## DISCUSSION

This large-scale meta-analysis of the vertical distribution of *C. finmarchicus* during the productive period synthesizes findings from earlier studies and confirms the copepod’s significant adaptability is thriving under various environmental conditions (Pedersen and Tande, 1992). Nevertheless, we found thresholds in temperature and salinity at which vertical distributions were significantly different, indicating a differential behavior in regions with these conditions. The investigated data set contains data points from the entire North Atlantic from 1971 to 2018 (Fig. 1), where high-latitude and coastal regions are well-represented while central oceanic regions are not. Region was included in all the statistical models fitted to the large data set (Table II); however, we did not focus on regional differences in this study but instead tried to discern common, stage-specific responses to environmental factors across the North Atlantic. Below we will discuss the role of environmental variables shaping the vertical distribution of *C. finmarchicus* developmental stages in detail.

### Stage-specific vertical distribution

In this meta-analysis, generally younger copepodites were distributed shallower than older stages. For older developmental stages, these stage-specific differences in WMDs are broadly consistent with the depth distribution observed in the Norwegian and Greenland Seas. There, CIVs predominantly occupied the upper 50 m, while CVs and adults were mostly found in the upper 80 m (Dale and Kaartvedt, 2000). Younger developmental stages (ES), on the other hand, were generally found slightly deeper in our study (WMD_ES_ = 37 m) than in the study by Dale and Kaartvedt (2000), which observed young stages in the upper 30 m. This difference might be due to the broader depth strata included in our meta-analysis, which may result in deeper WMDs estimate. Stage-specific habitat selection is viewed as balancing the risk of predation with resource demands, both of which increase with size and hence with developmental stage (Fiksen and Carlotti, 1998; Bandara *et al.,* 2018). *In situ* studies have confirmed the vertical segregation of different developmental stages across the North Atlantic, clearly indicating that copepodid stages respond differently to environmental factors (e.g. Irigoien, 2000; Basedow *et al.,* 2010). Our observed stage-specific variation in the vertical distribution of *C. finmarchicus* during the feeding months highlights the importance of considering the ontogeny and seasonal environmental dynamics to understand drivers of the vertical distribution. We will address specific environmental effects in the following sections.

### Effect of environmental factors on vertical distribution

The WMD of *C. finmarchicus* was consistently shallower in colder waters compared to warmer waters. At colder temperatures, copepods were concentrated in a shallow layer, in warmer waters they remained also shallower except for the early developmental stages, which were deeper. Part of this was caused by seasonal differences: WMD tended to be shallower in spring than in summer across all developmental stages. However, in spring all stages stayed shallower in the water column at colder temperatures. Similarly, Gaard *et al.* (2008), observed shallower positions of *C. finmarchicus* in colder areas and related that to the avoidance of warmer temperatures in their study across the North Atlantic ridge down to ca. 40^o^N. In our meta-analysis from a wider area, the shallowest distributions were observed at temperatures < 1°C, which cannot be explained by copepods trying to stay in waters around their temperature optimum. Colder temperatures delay gonad maturation and egg production, as well as development of *C. finmarchicus* (Niehoff *et al.,* 2000; Campbell *et al.,* 2001; Møller *et al.,* 2012; Kvile *et al.,* 2022). The observed shallower distribution with colder temperatures might be partly explained by a prolonged development time, compelling the copepods to stay shallower for a prolonged time. In contrast, higher temperatures may promote an earlier descent from surface waters (Grote *et al.,* 2015; Häfker *et al.,* 2018). Combined, the meta-analysis indicates that all stages of *C. finmarchicus* in the coldest areas of its distribution may need to stay longer in the surface layer to fulfill development. However, in the warmest areas only early stages remain deeper, likely to avoid warm surface waters, while older stages stay shallower, potentially influenced by other environmental factors, such as salinity, Chl.-*a* or light.

Salinity was another significant factor predicting WMD of *C. finmarchicus*. However, describing clear vertical distribution patterns based on salinity was challenging. This difficulty arises from the absence of discernible patterns within specific salinity intervals or across various developmental stages. Part of the explanation for these discrepancies can be attributed to the diversity of environments encompassed in this large-scale study. Fjord stations, for example, often present lower salinity levels and significant freshwater input, which might have influenced our predictions. *C. finmarchicus* populations thrive under a large seasonal variation in salinity (Skreslet *et al.,* 2000). While the meta-analysis does indicate an effect of salinity on the vertical distribution of *C. finmarchicus*, detailed studies are needed to disentangle the specific effect.

The meta-analysis confirms the important role of the phytoplankton spring bloom in the life cycle and development of *C. finmarchicus*. Recently it has been shown that *C. finmarchicus* has adopted different life histories in the different basins of the North Atlantic, with respect to resource allocation to eggs and lipids (Jónasdóttir *et al.,* 2022). In this meta-analysis, however, throughout the North Atlantic as a whole abundances of adults were highest during the peak of the bloom, while total abundances were highest one month after the bloom. Furthermore, during the peak of the bloom all stages were observed in the upper 50 m. Although the copepods might allocate resources differently in the different living areas, this analysis corroborates the classic understanding of *C. finmarchicus* as a predominantly herbivorous species with a life cycle tightly coupled to the phytoplankton bloom (Tande, 1982; Broms and Melle, 2007). At Chl.-*a* concentrations > 1.5 mg m^−3^, CIV and adults occurred significantly closer to the surface compared to lower Chl.-*a* concentrations. CV copepodites and adults obtain maximum feeding rates at 1.5–2 mg Chl.-*a* m^−3^, and might seek these higher Chl.-*a* concentrations to obtain highest food intake (Tande and Båmstedt, 1985; Basedow *et al.,* 2010). We did observe younger stages in the upper 50 m during the bloom, but did not find a significant effect of Chl.-*a* concentration on the vertical distribution of these stages. The general occurrence in the upper, food-rich layer is in line with the importance of the phytoplankton spring bloom for the growth potential of young stages (Broms and Melle, 2007; Reygondeau and Beaugrand, 2011). On the other hand, the lower demand for resources at younger developmental stages may result in these stages not seeking water layers with higher concentrations of resources (phytoplankton), which may explain why we did not observe a significant effect of Chl.-*a* concentration on the distribution of these stages in the water column. A study in the Subarctic Norwegian that sampled *Calanus* with high spatial resolution found developmental stages CII-CIII deeper in the water column, at around 30 m, when surface Chl.-*a* concentrations were high (Basedow *et al.,* 2010). This was explained by either larger and/or toxic algae not suitable for these developmental stages or surface predators feeding on these stages. The coarser resolution in this meta-analysis did not allow for detecting such small-scale vertical changes in the vertical segregation of copepodid stages. Due to missing Chl.-*a* data from many sampling points in this meta-analysis, we utilized ocean color remote sensing to detect surface blooms. Sub-surface phytoplankton blooms are not detected by satellites, but can be important for the development of older copepodites (Møller *et al.,* 2018). This study revealed stage-specific responses to the phytoplankton spring bloom throughout the North Atlantic. We recommend to measure and report Chl.-*a* concentrations in future studies, to be able to detect specific responses of copepodid stages to Chl.-*a*, as one of the best proxies of potential *C. finmarchicus* staple food.

Finally, we found that time of day was a significant factor explaining the vertical position of copepods in the water column; however, irradiance was not. This indicates that our irradiance calculations might not have been accurate enough. Light attenuation equations do not consider the shading effect by phytoplankton and other particles. Furthermore, inaccuracies in the measurements of cloud cover likely contributed to inaccurate irradiance values. The ERA5 archive data resolution is 6 hours, while cloud cover can change in a matter of minutes. However, even *in situ* light measurements failed to predict the WMD of *Calanus* spp. in a study by Lindegren *et al.* (2020). This could indicate that the observed effect, i.e. older copepodites staying slightly deeper with increasing time of day, is driven by an internal clock. Day length has previously been suggested to influence the circadian clock of *C. finmarchicus* (Häfker *et al.,* 2018). It is noteworthy, however, that both temperature and Chl.-*a* had a stronger influence than time of day on the vertical distribution of *C. finmarchicus*.

### Limitations and assumptions of the meta-analysis

Incorporating data from 1971 to the present meant including data collected with different sampling methodologies over time. Different sampling devices with different mesh sizes were used in the dataset depending on which institute and project was sampling. In the final GAM models that best explained variance in copepod vertical distribution, net type and mesh size were retained. This allowed to investigate the effect of environmental factors taking into account the differences in sampling method. Standardizing the datasets from the large area and time was not possible without making assumptions. We interpolated vertical profiles of copepod abundances to allow for comparisons with environmental factors. For this we assumed that copepods were equally distributed within the depth strata sampled, which most certainly is not true. Profiles with finer resolution are inherently more accurate than those with broader depth strata. While copepod distribution may have been uneven, the resolution of the data is fixed, as it is based on net catches. Nevertheless, we still use the WMD as it offers a more straightforward method for illustrating the species’ preferred depth. Focusing the analysis on the upper 200 m of the water column was necessary to target active individuals during spring and summer. We acknowledge that some individuals may have been captured on their descent, or in shallower environments where they could hibernate at 200 m. However, less than 30% of the stations included in this study had bottom depths < 200 m and we consider that the effect of potentially overwintering individuals will not have significantly influenced our results.

We focused on environmental factors explaining stage-specific differences in vertical distribution, however, could not include predation in our analyses due to missing data. Predation pressure can influence vertical distribution by altering key behaviors, such as the timing of reproduction e.g. due to inducing early spawning strategy (Kaartvedt, 2000). Additionally, their vertical position can be influenced by a trade-off between feeding and avoiding visual predators, balancing the need to find food with the advantage of staying in darker waters (Aksnes *et al.,* 2004; Varpe and Fiksen, 2010). Accordingly, zooplankton biomass in the upper 100 m has been found to be low in the presence of capelin, suggesting rapid prey depletion (Hassel *et al.,* 1991). Furthermore, as previously suggested predation pressure can also shape stage-specific vertical distribution patterns, as older stages are larger and may tend to inhabit deeper waters to reduce predation risk, while smaller, less visible younger stages remain in shallower depths (Daase *et al.,* 2008). A stage’s distribution could also be associated with competition between stages, as different developmental stages may compete for resources. Understanding how predation interacts with environmental factors is crucial for explaining stage-specific depth distribution and the ecological dynamics of zooplankton, as well as seasonal population dynamics. Increased mortality may be linked to a decline in the abundance of preferred prey or greater predation pressure, which tends to be higher in summer. Summer populations, which typically have shorter life cycles, may also face higher predation risk and increased mortality due to stronger competition, as shown for the small copepod species *Oithona similis* (Dvoretsky, 2012). We recommend that efforts are made to combine, whenever possible, studies of ecosystem components at different trophic levels (i.e. zooplankton prey and pelagic fish predator, or even better, phytoplankton, zooplankton, fish) for better understanding of organism specific stage-specific depth distribution, important for comprehending the ecological dynamics of individual species or species assemblages.

### *C. finmarchicus* in a changing ocean

The comprehensiveness of our research can provide a baseline to study how environmental factors will shape future vertical distributions. *C. finmarchicus* is widely distributed in habitats across the North Atlantic and Subarctic (Broms *et al.,* 2009; Melle *et al.,* 2014). This meta-analysis confirms that high abundances were observed in all regions across the North Atlantic, at various environmental conditions. The ability of *C. finmarchicus* to adapt to a range of environmental conditions could be a key factor in their ecological success and this ecological flexibility might also allow for an adaptation to future changes (Trudnowska *et al.,* 2020a; Balazy *et al.,* 2021). Additionally, younger stages of a new *Calanus* generation (copepodites CI–CIII) have been found to tolerate a noticeably wider range of temperatures, making them less stenothermal than older stages. This greater tolerance may influence their distribution and survival in a changing environment (Pertsova and Kosobokova, 2010). Global warming and the associated rise in temperatures likely will impact the timing, composition and magnitude of phytoplankton spring blooms (Sommer and Lengfellner, 2008). Our results show that these predicted changes in phytoplankton blooms will have direct effects on *C. finmarchicus* vertical distribution. In turn, changes in the vertical distribution of *C. finmarchicus*, such as the deeper positioning of older stages after the bloom, might directly affect predators due to changes in visual detecting their prey (Langbehn and Varpe, 2017). Across the North Atlantic, in a variety of habitats, *C. finmarchicus* was found in surface waters during the peak spring bloom. That is, the meta-analysis indicates that *C. finmarchicus* was able to match with the phytoplankton spring bloom across a range of conditions. In contrast, a mismatch of primary producers and *C. glacialis* has been proposed with changes in the seasonal development of ice algae and phytoplankton blooms (Søreide *et al.,* 2010,), and recently a mismatch between phytoplankton and *C. finmarchicus* was observed at its northern range of distribution (Renaud *et al.,* 2024). This highlights the importance of closely monitoring the development of the species in coming years. Data over larger temporal and spatial scale like in this study are useful to detect match/mismatch scenarios. The recent development in detecting not only phytoplankton but also *C. finmarchicus* by ocean color remote sensing facilitates this task considerably (McCarry *et al.,* 2023).

## CONCLUSIONS

This summary using statistical GAM models of a large amount of data on the spring and summer occurrence of *C. finmarchicus*, including its developmental stages, complements and enhances previous research efforts. The position of specific copepodite stages of this species in the upper water column varied depending on the developmental stage and time of the year. It was additionally regulated by surface concentration of Chl.-*a* and temperature. A significant finding was the strong correlation between surface Chl.-*a* peaks and shallower vertical distribution across all developmental stages highlighting the importance of the phytoplankton bloom in shaping the species distribution. We also found a significant effect of temperature on the vertical distribution of developmental stages. In warmer scenarios, early stages were likely avoiding warmer surface waters and were observed in deeper waters. In colder waters, at the northern edge of distribution, copepods occupied shallower depths.

Although not trivial, we recommend that efforts are made to sample predators in conjunction with zooplankton and environmental factors. Information on predators is often missing in zooplankton studies, which hampers understanding their role in the marine environment, for example in shaping vertical distributions of *C. finmarchicus*. Here, acoustic measurements at different frequencies might be very helpful to understand how predation interacts with environmental factors to influence depth distribution (Flores *et al.,* 2023). The vertical distribution of zooplankton, and of *C. finmarchicus* in particular, drives biogeochemical cycles in addition to food web ecology structure. To detect interactions between primary producers and grazers on relevant scales, we recommend aiming at sampling the upper 100 m with higher spatial resolution than is commonly used, e.g. 10 m depth layers. Future studies on the vertical distribution of *C. finmarchicus* should include Chl.-*a* concentration measurements in the water column. To be able to detect potential stage-specific responses to subsurface Chl.-*a* maxima. Global warming might increase the importance of subsurface phytoplankton blooms, further highlighting the importance of having suitable measurements from the subsurface water column (Viljoen *et al.,* 2024).

## Supplementary Material

figA1_fbaf019

figA2_fbaf019

figA3_fbaf019

JPR_supplementary_Chamorro_fbaf019

Supplementary_fig_captions_fbaf019

## Data Availability

The data used for this study are cited in the article, further inquiries can be directed to the corresponding author.
