# Supplementary figures and images for: Meta-analysis of stage-specific Calanus finmarchicus vertical distribution in relation to hydrography and chlorophyll in the North Atlantic
Source: J Plankton Res. 2025 Jun 21;47(4):fbaf019. doi: 10.1093/plankt/fbaf019 (PMC12205936; doi:10.1093/plankt/fbaf019)

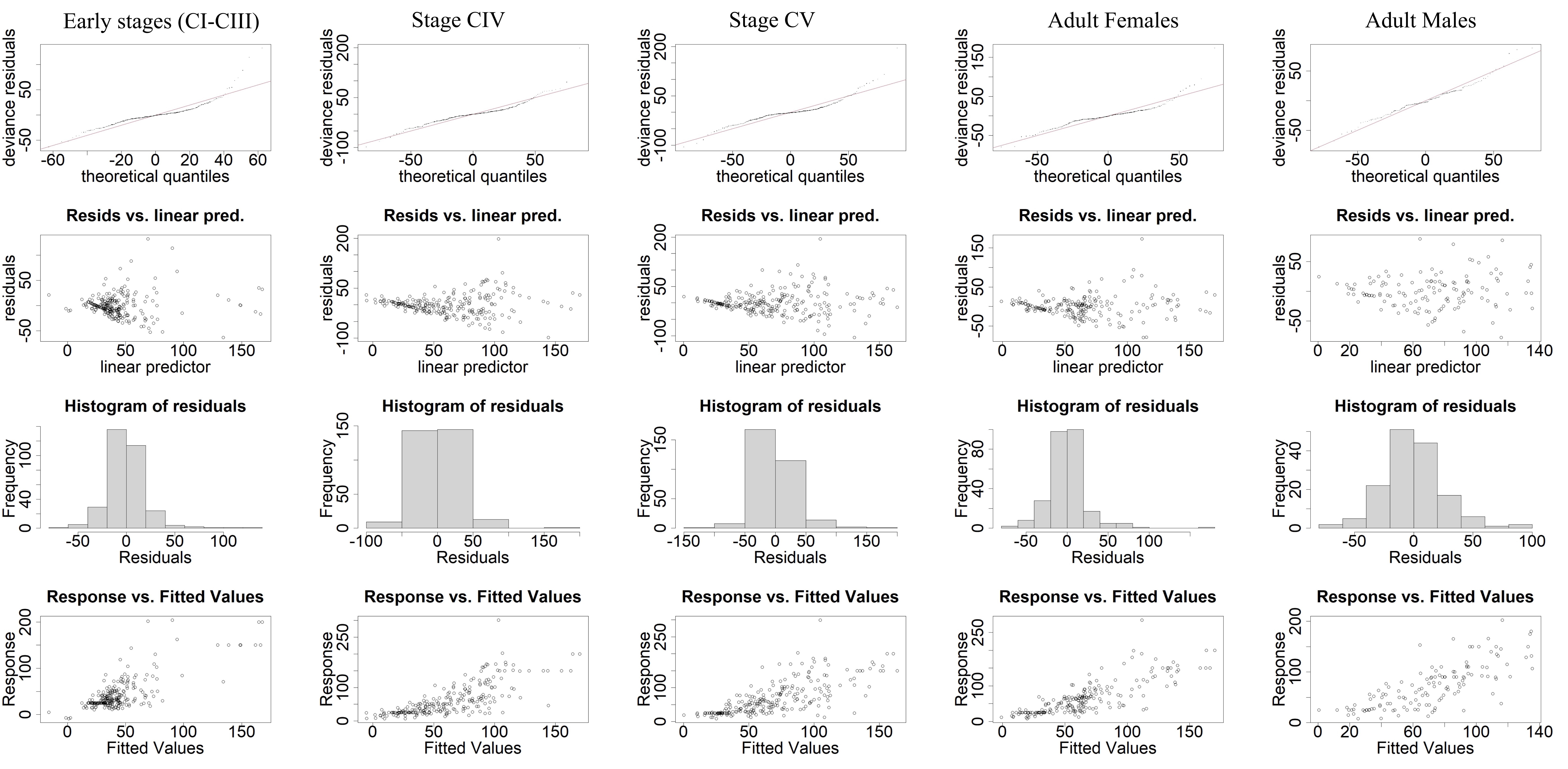

Supplement: figA1_fbaf019 [file figa1_fbaf019.jpeg]

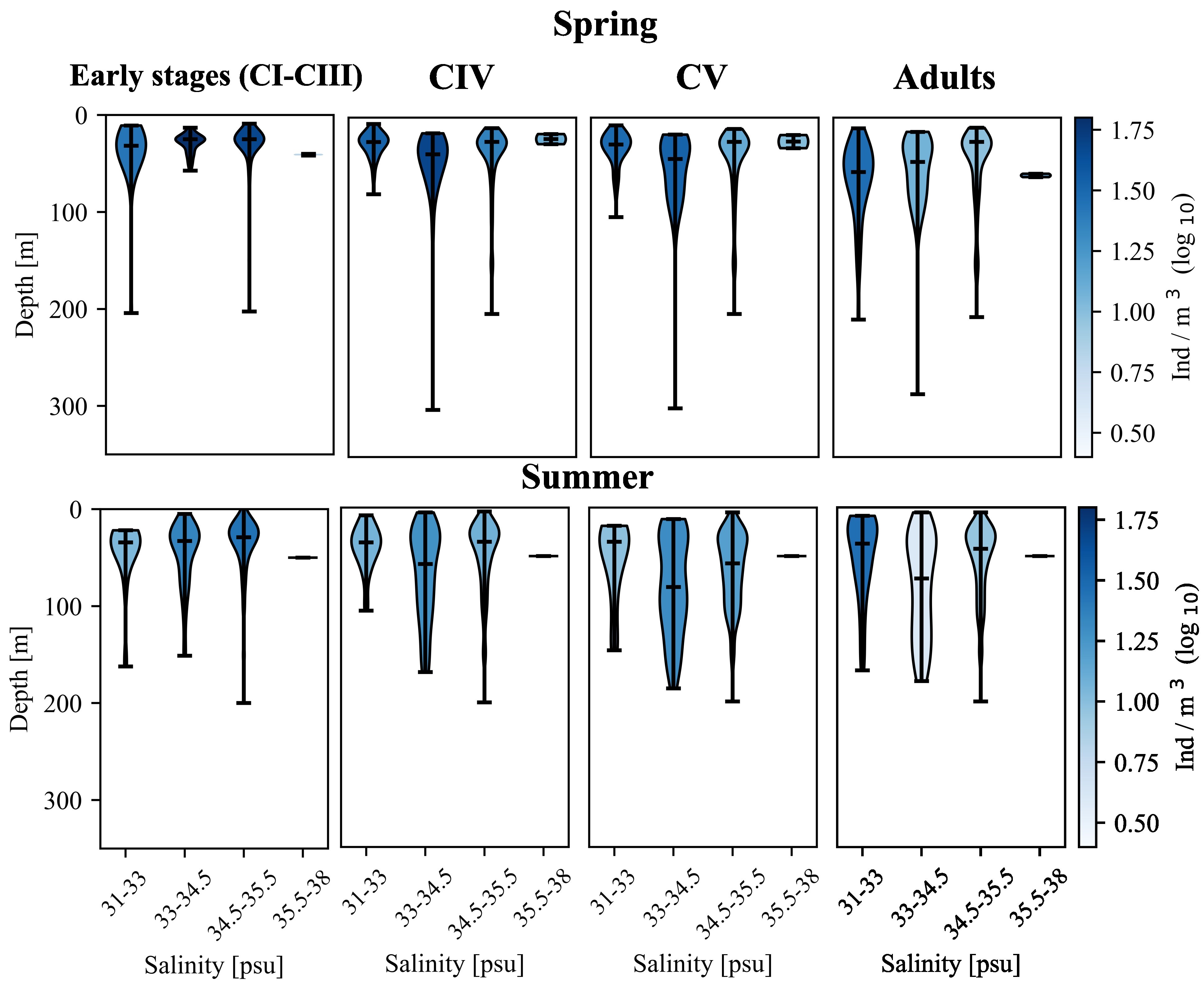

Supplement: figA2_fbaf019 [file figa2_fbaf019.jpeg]

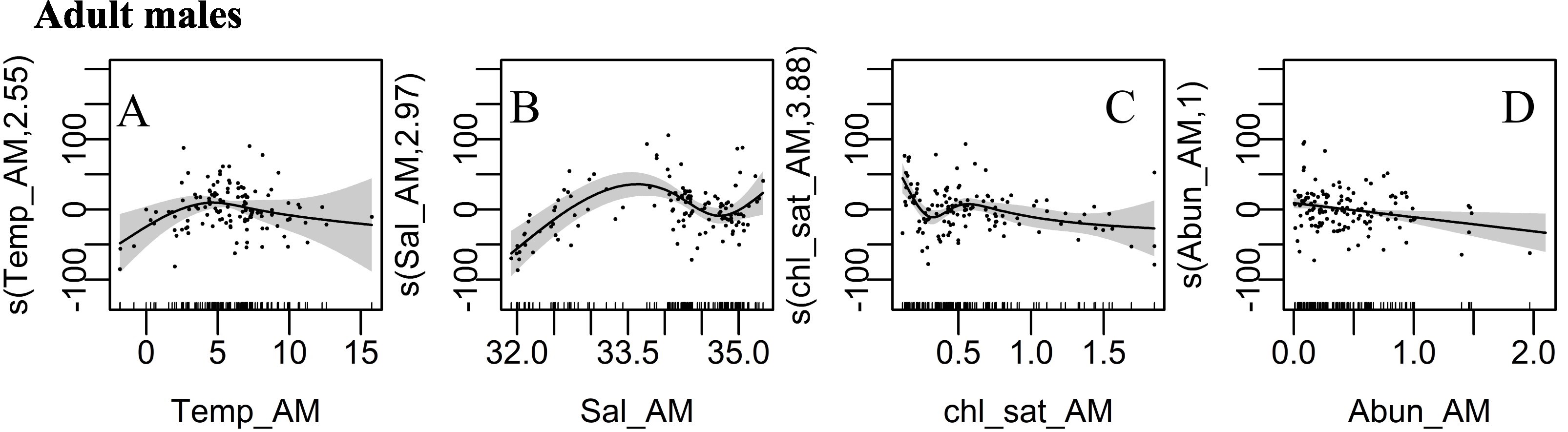

Supplement: figA3_fbaf019 [file figa3_fbaf019.jpeg]
