# Supplementary material for: Meta-analysis of stage-specific Calanus finmarchicus vertical distribution in relation to hydrography and chlorophyll in the North Atlantic
Source: J Plankton Res. 2025 Jun 21;47(4):fbaf019. doi: 10.1093/plankt/fbaf019 (PMC12205936; doi:10.1093/plankt/fbaf019)
Supplement: JPR_supplementary_Chamorro_fbaf019 [file jpr_supplementary_chamorro_fbaf019.docx]

SUPPLEMENTARY MATERIAL

Table A1: Model selection. Contribution of covariates to the generalized additive mixed model fitted with data from different development stages: early stages (ES), CIV, CV, adult females (AF) and adult males (AM). The first column shows the models fitted for the different development stages where the covariates are marked with their significance levels: 0 ‘***’ 0.001 ‘**’ 0.01 ‘*’ 0.05 ‘^●^’ 0.1. The second columns show the value of Akaike’s Information Criterion (AIC) for each model and the third column the adjusted coefficient of determination (R^2^ adjusted). The last column shows the deviance explained by each model. Grey shadow stands for the model that better performed the data based on AIC and covariates significance.

| **Models fitted for each stage** | **AIC** | **R^2^** | **Deviance explained** |
| --- | --- | --- | --- |
| **ES** |  |  |  |
| WMD_ES~s(Temp***)+s(sal***)+s(abun)+s(time)+s(Irr)+s(Chl_peak_days)+s(Chlsat)+MarineRegion+Month+s(Net_ES)+ s(Mesh_ES) + s(Net_ES, Mesh_ES**) | 2895 | 0.54 | 57.3% |
| WMD_ES~s(Temp***)+s(sal***)+s(abun)+s(time)+s(Irr)+s(Chl_peak_days)+Marine Region+Month+s(Net_ES)+ s(Mesh_ES) + s(Net_ES, Mesh_ES ***) | 2894 | 0.54 | 57.2% |
| WMD_ES~s(Temp***)+s(sal***)+s(abun)+s(time)+s(Irr)+Marine Region+Month+s(Net_ES)+ s(Mesh_ES) + s(Net_ES, Mesh_ES ***) | 2893 | 0.54 | 57.1% |
| WMD_ES~s(Temp***)+s(sal***)+s(abun)+s(time^●^)+Marine Region+Month+ s(Net_ES)+ s(Mesh_ES) + s(Net_ES, Mesh_ES ***) | 2891 | 0.54 | 56.9% |
| WMD_ES~s(Temp***)+s(sal***)+s(time ^●^) +Marine Region+Month+ s(Net_ES)+ s(Mesh_ES) + s(Net_ES, Mesh_ES ***) | 2890 | 0.54 | 56.9% |
| WMD_ES~s(Temp***)+s(sal***)+Marine Region+Month + s(Net_ES)+ s(Mesh_ES) + s(Net_ES, Mesh_ES *) | 2892 | 0.54 | 56.1% |
| WMD_ES~s(Temp***)+Marine Region+Month + s(Net_ES)+ s(Mesh_ES) + s(Net_ES, Mesh_ES ***) | 2907 | 0.51 | 53.4% |
| **CIV** |  |  |  |
| WMD_CIV~s(Temp***)+s(sal***)+s(Chlsat*)+s(time*)+ s(abun^●^) +s(Chl_peak_days)+s(Irr)+Marine Region+Month + s(Net_ES)+ s(Mesh_ES) + s(Net_ES, Mesh_ES ***) | 3018 | 0.55 | 58.7% |
| WMD_CIV~s(Temp***)+s(sal***)+s(Chlsat*)+s(time**)+ s(abun●) +s(Chl_peak_days)+Marine Region+Month + s(Net_ES)+ s(Mesh_ES) + s(Net_ES, Mesh_ES ***) | 3016 | 0.55 | 58.7% |
| WMD_CIV~s(Temp***)+s(sal***)+s(Chlsat*)+s(time**)+ s(abun●) +Marine Region+Month+ s(Net_ES)+ s(Mesh_ES) + s(Net_ES, Mesh_ES ***) | 3015 | 0.55 | 58.4% |
| WMD_CIV~s(Temp***)+s(sal***)+s(Chlsat●)+s(time*)+Marine Region+Month + s(Net_ES)+ s(Mesh_ES) + s(Net_ES, Mesh_ES ***) | 3017 | 0.55 | 57.9% |
| WMD_CIV~s(Temp***)+s(sal***)+s(time*)+Marine Region+Month + s(Net_ES)+ s(Mesh_ES) + s(Net_ES, Mesh_ES ***) | 3018 | 0.54 | 57.5% |
| WMD_CIV~s(Temp***)+s(sal***)+Marine Region+Month + s(Net_ES)+ s(Mesh_ES) + s(Net_ES, Mesh_ES ***) | 3023 | 0.53 | 56.3% |
| WMD_CIV~s(Temp***)+Marine Region+Month + s(Net_ES)+ s(Mesh_ES) + s(Net_ES, Mesh_ES ***) | 3058 | 0.48 | 50.5% |
| **CV** |  |  |  |
| WMD_CV~s(Temp●)+s(sal***)+s(Chlsat)+s(time*)+s(abun)+s(Chl_peak_days)+s(Irr)+Marine Region+Month + s(Net_ES***)+ s(Mesh_ES***) | 3035 | 0.55 | 58.9% |
| WMD_CV~s(Temp●)+s(sal***)+s(Chlsat ●)+s(time*)+s(abun)+s(Irr)+Marine Region+Month + s(Net_ES***)+ s(Mesh_ES***) | 3033 | 0.55 | 58.7% |
| WMD_CV~s(Temp*)+s(sal***)+s(Chlsat●)+s(time*)+s(abun)+Marine Region+Month + s(Net_ES***)+ s(Mesh_ES***) | 3031 | 0.55 | 58.7% |
| WMD_CV~s (Temp●) +s(sal***)+s(Chlsat ●)+s(time*)+Marine Region+Month + s(Net_ES***)+ s(Mesh_ES***) | 3030 | 0.55 | 58.6% |
| WMD_CV~s(Temp*)+s(sal***)+s(time ●)+Marine Region+Month +s(Net_ES***)+ s(Mesh_ES***) | 3031 | 0.55 | 58.2% |
| WMD_CV~s (Temp●) +s(sal***)+Marine Region+Month +s(Net_ES***)+ s(Mesh_ES***) | 3035 | 0.54 | 57.2% |
| WMD_CV~s(sal***)+Marine Region+Month +s(Net_ES***)+ s(Mesh_ES***) | 3038 | 0.53 | 56.2% |
| **AF** |  |  |  |
| WMD_AF~s(Temp**)+s(sal***)+s(Chlsat)+s(time**)+s(abun)+s(Chl_peak_days)+s(Irr)+Marine Region+Month+s(Net_ES)+ s(Mesh_ES ●) + s(Net_ES, Mesh_ES *) | 2522 | 0.63 | 66.8% |
| WMD_AF~s(Temp**)+s(sal***)+s(Chlsat)+s(time***)+s(abun*)+s(Chl_peak_days)+Marine Region+Month +s(Net_ES)+ s(Mesh_ES ●) + s(Net_ES, Mesh_ES *) | 2520 | 0.63 | 66.8% |
| WMD_AF~s(Temp**)+s(sal***)+s(Chlsat)+s(time***)+s(abun)+Marine Region+Month +s(Net_ES) + s(Mesh_ES ●) + s(Net_ES, Mesh_ES *) | 2519 | 0.63 | 66.7% |
| WMD_AF~s(Temp*)+s(sal***)+s(time***)+s(abun)+Marine Region+Month +s(Net_ES) + s(Mesh_ES ●) + s(Net_ES, Mesh_ES *) | 2518 | 0.64 | 66.5% |
| WMD_AF~s(Temp*)+s(sal***)+s(time***)+Marine Region+Month +s(Net_ES) + s(Mesh_ES*) + s(Net_ES, Mesh_ES *) | 2519 | 0.63 | 66.1% |
| WMD_AF~s(Time**)+s(sal***)+Marine Region+Month +s(Net_ES) + s(Mesh_ES **) + s(Net_ES, Mesh_ES ) | 2523 | 0.63 | 65.2% |
| WMD_AF~s(sal***)+Marine Region+Month +s(Net_ES) + s(Mesh_ES **) + s(Net_ES, Mesh_ES ) | 2535 | 0.60 | 63% |
| **AM** |  |  |  |
| WMD_AM~s(Temp***)+s(sal***)+s(Chlsat**)+s(time^●^)+s(abun*)+s(Chl_peak_days)+s(Irr)+Marine Region+Month + s(Net_AM, Mesh_AM ***) | 1490 | 0.45 | 53% |
| WMD_AM~s(Temp***)+s(sal***)+s(Chlsat**)+s(time*)+s(abun*)+s(Chl_peak_days)+Marine Region+Month+ s(Net_AM, Mesh_AM ***) | 1488 | 0.45 | 53% |
| WMD_AM~s(Temp***)+s(sal***)+s(Chlsat**)+s(time^●^)+s(abun*)+Marine Region+Month+s(Net_AM, Mesh_AM ***) | 1487 | 0.45 | 52.4% |
| WMD_AM~s(Temp***)+s(sal***)+s(Chlsat**)+s(abun**)+Marine Region+Month +s(Net_AM, Mesh_AM ***) | 1490 | 0.44 | 50.7% |
| WMD_AM~s(Temp**)+s(sal***)+s(Chlsat**)+Marine Region+Month +s(Net_AM, Mesh_AM ***) | 1497 | 0.41 | 47.8% |
| WMD_AM~s(Temp**)+s(sal***)+Marine Region+Month +s(Net_AM, Mesh_AM ***) | 1506 | 0.37 | 43.6% |
| WMD_AM~s(sal***)+Marine Region+Month | 1514 | 0.32 | 39.1% |
