# Supplementary material for: Meta-analysis of stage-specific Calanus finmarchicus vertical distribution in relation to hydrography and chlorophyll in the North Atlantic
Source: J Plankton Res. 2025 Jun 21;47(4):fbaf019. doi: 10.1093/plankt/fbaf019 (PMC12205936; doi:10.1093/plankt/fbaf019)
Supplement: Supplementary_fig_captions_fbaf019 [file supplementary_fig_captions_fbaf019.docx]

Fig. A1. Generalized additive model (GAM) check plots for the five models presented. Residual plots for checking the GAM model ﬁtting process.

Fig. A2. Violin plot representation of the WMD and mean abundances of *C. finmarchicus* stages by mean salinity intervals within the upper water column in spring and summer. The black/grey middle horizontal line denotes median values, while the upper and lower ones represent maximum and minimum respectively.

Fig. A3. Effect of significant variables (temperature, salinity, surface chlorophyll-*a,* and abundance) on the vertical distribution (weighted mean depth) of adult males *C. finmarchicus*. Partial effect (solid line), 95% confidence intervals (shadow area) and residuals (grey dots) are depicted as well. Data were sampled in the North Atlantic during spring and summer following different methods (See Table 1).
